# Extensive Suppuration, and Death, from the Prick of a Pin

**Published:** 1829-04-01

**Authors:** 


XLIV.

### EXTENSIVE SUPPURATION AND DEATH SUCCEEDING THE PRICK OF A PIN.*

Mr. Cunninghame has published, in our respected Glasgow contemporary, an interesting case of this kind, differing from those which not uncommonly occur, inasmuch as no suppuration would seem to have taken place in the immediate neighbourhood of the puncture. The case, indeed, seems rather to resemble those instances of "purulent depôts" which are now attracting such general attention. We subjoin the particulars, in order that our readers may judge for themselves.

A woman, ætatis 56, of spare and delicate habit, who had always enjoyed a tolerable share of health, but never could bear much exertion or fatigue, pricked the point of her fore-finger with a pin whilst washing or dressing some clothes. She continued her work till the evening, but, during the night, felt hot and restless, with pain and swelling of the hand and finger. Poultices were applied, but the hand became worse, and our author was requested to see her on the third day after the occurrence of the accident. There was much febrile excitement, with pain and swelling in the finger and hand, but no pus was as yet collected. With the view, however, of relieving tension, an incision was made to the bone, in the spot where the injury had been received, a cold lotion was ordered, and a cathartic administered internally. This practice was persevered in for a while, without any mitigation of symptoms; the incision in the finger began to discharge a thin, unhealthy-looking pus; the absorbents inflamed as high as the elbow, but no tension existed higher than the wrist. Leeches, aperients, and diuretics were employed, but the unfavourable symptoms still increased—the constitutional affection grew greater—the pulse was quick and small—debility extreme—retching and vomiting constant—appetite gone—pain in the arm when moved excruciating—discharge from the incision black and fetid—first phalanx of the finger insensible, and the bone felt in a half-dissolved state—no fluctuation in any part of the arm, nor enlargement of the axillary glands. Another incision was made over the second phalanx of the same finger, more leeches were applied, turpentine liniment used to the part, and the arm fomented with decoction of poppies and solution of the acetate of lead. Sulphate of quinine and sulphuric acid were also exhibited internally. No improvement whatever took place, and, on the 12th day, the phalanx, being completely sphacelated, was removed. The glands in the axilla were swelled and painful, but there was no indication of suppuration having taken place, either there or in the arm. The irritability of the stomach continued, and the patient was unable to swallow any medicine; but wine and beef-tea were ordered.

On the 17th day, fluctuation was distinct over the inferior angle of the scapula, where an opening was made, and two tea-cupfuls of thin fetid pus let out. Great detachment was found to have taken place, and the matter had burrowed extensively down the lateral and back parts of the chest, though an opening in the axilla discovered no "track" extending down the arm.

* Glasgow Med. Journal, No. V.

"It was now evident, from the very debilitated state of the patient—from her inability to take nourishment of any kind, and from the great extent of suppurating surface, that but small hopes could be entertained of her recovery. Counter-openings were made at the most depending parts of the abscess, stimulating injections, pressure, and bandaging were all had recouse to, while tonics and cordials, in every form, were tried, but without in the least improving the quality of the secretions, or causing the slightest disposition to adhesive inflammation. Towards the end of this poor woman's life, large shreds of cellular membrane came away by the wounds, and, for a day or two previous to her death, some parts of the ribs were quite bared of their coverings. She died on the thirty-eighth day from the injury, and the eighteenth from the opening of the abscess."

The author appends some remarks to the case, with the view of shewing that the fatal consequences of so trivial an injury were owing to an idiosyncrasy of his patient's constitution, and predisposition to irritation. However this may be, the case, as we stated before, resembles, in many of its features, the purulent deposits in various parts of the body, and differs materially from the ordinary cases of suppuration after pin-pricks, or other punctured wounds. The length of time that the patient lived (thirty-eight days after the accident) is strongly in favour of the former supposition.

